# Study protocol of the quasi-experimental evaluation of “KEIGAAF”: a context-based physical activity and nutrition intervention for primary school children

**DOI:** 10.1186/s12889-018-5764-3

**Published:** 2018-07-06

**Authors:** S. R. B. Verjans-Janssen, Dave H. H. Van Kann, Sanne M. P. L. Gerards, Steven B. Vos, Maria W. J. Jansen, Stef P. J. Kremers

**Affiliations:** 1 Department of Health Promotion, NUTRIM School of Nutrition and Translational Research in Metabolism, Maastricht University, PO Box 616, Maastricht, The Netherlands; 2 School of Sport Studies, Fontys University of Applied Sciences, Eindhoven, The Netherlands; 3 Department of Industrial Design, Eindhoven University of Technology, Eindhoven, The Netherlands; 4 Academic Collaborative Center for Public Health, Public Health Service South-Limburg, Heerlen, The Netherlands; 5 Department of Health Services Research, Maastricht University, CAPHRI Care and Public Health Research Institute, Maastricht, The Netherlands

**Keywords:** Physical activity, Sedentary behavior, Nutrition, Children, Primary school, Family, Intervention

## Abstract

**Background:**

The environment affects children’s energy balance-related behaviors to a considerable extent. A context-based physical activity and nutrition school- and family-based intervention, named KEIGAAF, is being implemented in low socio-economic neighborhoods in Eindhoven, The Netherlands. The aim of this study was to investigate: 1) the effectiveness of the KEIGAAF intervention on BMI z-score, waist circumference, physical activity, sedentary behavior, nutrition behavior, and physical fitness of primary school children, and 2) the process related to the implementation of the intervention.

**Methods:**

A quasi-experimental, controlled study with eight intervention schools and three control schools was conducted. The KEIGAAF intervention consists of a combined top-down and bottom-up school intervention: a steering committee developed the general KEIGAAF principles (top-down), and in accordance with these principles, KEIGAAF working groups subsequently develop and implement the intervention in their local context (bottom-up). Parents are also invited to participate in a family-based parenting program, i.e., Triple P Lifestyle. Children aged 7 to 10 years old (grades 4 to 6 in the Netherlands) are included in the study. Effect evaluation data is collected at baseline, after one year, and after two years by using a child questionnaire, accelerometers, anthropometry, a physical fitness test, and a parent questionnaire. A mixed methods approach is applied for the process evaluation: quantitative (checklists, questionnaires) and qualitative methods (observations, interviews) are used. To analyze intervention effectiveness, multilevel regression analyses will be conducted. Content analyses will be conducted on the qualitative process data.

**Discussion:**

Two important environmental settings, the school environment and the family environment, are simultaneously targeted in the KEIGAAF intervention. The combined top-down and bottom-up approach is expected to make the intervention an effective and sustainable version of the Health Promoting Schools framework. An elaborate process evaluation will be conducted alongside an effect evaluation in which multiple data collection sources (both qualitative and quantitative) are used.

**Trial registration:**

Dutch Trial Register NTR6716 (registration date 27/06/2017, retrospectively registered), METC163027, NL58554.068.16, Fonds NutsOhra project number 101.253.

**Electronic supplementary material:**

The online version of this article (10.1186/s12889-018-5764-3) contains supplementary material, which is available to authorized users.

## Background

A large number of Dutch children do not meet the national recommendations for physical activity and healthy nutrition behavior. In 2015, less than half of the children (48%) aged 4 to 12 years were moderately physically active at least 60 min per day, with activities aimed at improving or maintaining physical fitness being done at least two times per week [1]. Moreover, children in the same age category spent on average 7.3 h per day being sedentary [1, 2]. Compliance with Dutch healthy nutrition guidelines is even lower; for example, only 5% of children between the ages of 7 and 19 years met the Dutch guidelines for daily fruit consumption (2 pieces a day), and just 1% met those for vegetable consumption (250 g daily) [3]. Additionally, unhealthy foods are consumed in large quantities. For example, a study on the consumption of sugar-sweetened beverages among a sample of Dutch primary school children from diverse ethnic backgrounds showed a consumption level of 0.9 l per day [4]. This study shows that these unhealthy balance-related behaviors are more prevalent among children living in families with a low socio-economic position [5–9].

The environment has a great influence on children’s physical activity (PA), sedentary behavior (SB), and nutrition behavior (NB) - referred to jointly as energy balance-related behaviors (EBRBs) [10]. At the micro-environmental level, the family and school environment are important settings that influence children’s EBRBs [11], with the child’s parents exerting the most important influence on their children’s EBRBs [12–17]. For example, parents can affect their child’s EBRBs by applying certain parenting practices, like monitoring dietary intake [13]. The physical home environment also stimulates or discourages healthy EBRBs, e.g., by the availability of healthy or unhealthy foods at home [18].

On weekdays, the school environment heavily influences children’s PA [19]. The mode of transportation to and from school, physical activity during school recess (playtime), and physical education (PE) add to children’s levels of physical activity behavior on a school day [20]. On the other hand, the school environment generally facilitates sedentariness among children, since most educational activities are performed in a seated position [21]. Because the children often consume lunch, beverages, and snacks during school time, schools can also affect the children’s nutrition behavior. Even though Dutch primary school children bring their own beverages, snacks, and lunch to school, school nutrition policies (e.g., what children are allowed to bring to school) and healthy nutrition-promoting interventions can stimulate healthy nutrition behavior at school [22, 23].

Initiatives such as The Healthy School have been put in place to change children’s health behaviors via schools [24, 25]. In a more traditional approach, interventions are developed by the researchers or health promoters, based on theory and best practices. Subsequently, the interventions are implemented in a relatively top-down fashion: intervention implementers, e.g. school teachers or school staff, are required to adhere to the intervention protocol and implement the intervention as intended with strict fidelity [26]. In such a top-down approach, the complexity of school systems is not taken sufficiently into account [26]. Each school is different, and each context in which the school operates can be considered unique [27]. The effectiveness of a school-based intervention depends on multiple factors within this context, such as the characteristics of the community (e.g. socio-economic status), characteristics of the school staff, school needs and priorities, internal and external collaborations, the school’s physical environment, and its resources [27].

In a bottom-up approach, in which an intervention is developed by the users, in this case the schools, these contextual factors are taken into account, resulting in an intervention that meets the local needs and possibilities and is compatible with the school’s priorities [28–30]. This, in turn, encourages the school’s ownership of the intervention and enhances sustainability in the long-term [26, 28, 31]. A purely bottom-up approach may lead to the adoption and implementation of interventions that lack theoretical or empirical evidence regarding their impact on sustained behavior change [32]. Therefore, in the current project, a combined top-down and bottom-up approach is applied in which a school-based physical activity and nutrition intervention is developed and implemented by the school, local professionals, and parents, and guidelines about evidence are provided by a steering committee.

To ensure that not only the child’s school environment but also the parents are involved in the intervention, the parents are invited to participate in a family-based lifestyle parenting program. Family-based parenting interventions have proven to be effective in reducing overweight among children and improving their EBRBs [33, 34]. As it is unclear whether high-risk children benefit enough from school-based interventions targeted at all children [35, 36], this part of the project is focused on parents in need of additional support regarding lifestyle-related parenting. This may contribute to the reduction of health inequalities between healthy children and high-risk children [37, 38].

The overall aim of the context-based school- and family-based interventions is to improve the EBRBs of children living in low socio-economic neighborhoods by creating an environment that stimulates healthy EBRBs and discourages unhealthy EBRBs. The intervention, called KEIGAAF (‘KEIGAAF’ is a Dutch acronym and a local term referring to ‘*super cool*’), is based on two promising avenues: a school-based physical activity and nutrition intervention combining a top-down and bottom-up approach [39], and a family-based lifestyle coaching intervention [33]. In this paper, the development and implementation of the KEIGAAF intervention are elaborated. The study design is outlined which evaluates the intervention’s effectiveness on the PA behavior, sedentary behavior, nutrition behavior, weight status, and physical fitness of children aged 7 to 10 years living in low socio-economic neighborhoods (Additional file 1). To evaluate whether effects on children’s outcome measures are due to the intervention, a quasi-experimental controlled evaluation study will be conducted: the results of an intervention group will be compared with a control group consisting of children attending schools where the KEIGAAF intervention is not implemented.

A process evaluation will be conducted in addition to the effect evaluation. It is essential to look at intervention effectiveness in the context in which the intervention is implemented. An understanding of what is going on locally when implementing the intervention helps to clarify what works in what situation and for whom. This knowledge is essential when transferring effective EBRB-promoting intervention elements in the future to other situations [40]. Therefore, the second aim of this study is to evaluate the implementation of the KEIGAAF intervention (what is implemented and how is it implemented) and how contextual factors influence this implementation.

## Methods

### Study design

The aim of the KEIGAAF project is to design and test the effectiveness of the school- and family-based intervention on the primary outcome measure (BMI z-score) and the secondary outcome measures (PA behavior, sedentary behavior and nutrition behavior, physical fitness, and waist circumference) of children aged 7–10 years living in low socio-economic neighborhoods.

A convenience sample of intervention schools was recruited in Eindhoven, a relatively large city in the southern part of the Netherlands. Schools were eligible if: 1) they were located in a low socio-economic neighborhood; 2) they had no plans to merge with another school or to relocate in the coming three years, and if; 3) the school staff was willing to participate actively in KEIGAAF and to collaborate with parents and local partners, for instance, sports professionals, social workers, and/or municipality officers. The steering committee of the KEIGAAF project, consisting of researchers from Maastricht University and Fontys University of Applied Sciences (School of Sports Studies) and local partners from a school board, a sports support organization, and a social welfare organization, identified nine eligible schools. All nine schools were contacted and informed about the project, and eight schools agreed to participate. Because of the active participation of the intervention schools in the development and implementation of the intervention, no randomization was applied.

**Fig. 1:**
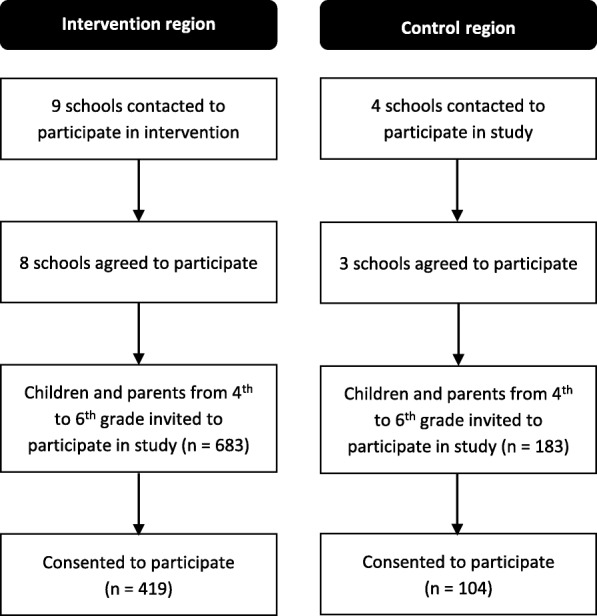
Study design

Control schools were recruited in Maastricht, a city comparable to Eindhoven based on the level of urbanization and the socio-economic status of the neighborhoods. Three schools agreed to participate in the study; these schools did not apply the KEIGAAF intervention (Fig. 1). Due to the distance between Eindhoven and Maastricht (approximately 90 km), a low risk of contamination is expected. KEIGAAF is funded by Fonds NutsOhra, project number 101.253, and the trial is registered as NTR6716. Ethical approval was obtained from the medical ethics committee of the Maastricht University Medical Centre (METC163027, national number: NL58554.068.16).

### Participants

In the Dutch education system, children attend first grade at age 4. For this study, all children from grades 4 to 6, thus between the age of 7 and 10 years, of the participating intervention and control schools were eligible for inclusion. The researcher visited the eligible classes of the participating schools and talked to the children about the study. The children were given written information about the study to take home to their parents. In the parental information letter, the parents were informed about this specific information moment. They were also given the opportunity to ask questions about the study of the researcher during planned school meetings. School principals posted a message about the study in the school newsletter and/or on the school website well in advance. Once the parents and the child decided to participate, both parents signed an informed consent. Of the eligible children, 60.4% (61.3% in the intervention group and 56.8% in the control group) consented to participate in the study.

Parents of children who attended schools with relatively high proportions of overweight and obesity as defined during baseline measures (March and April 2017) were invited to participate in Lifestyle Triple P, the family-based parenting program. This strategy was applied to prevent stigmatization. The parents were recruited via written information: a flyer was distributed among the parents, a message was posted in the school newsletter and/or on the school website, and a poster was displayed at the entrance of the school. In addition, the family-based intervention coaches and the school doctor personally recruited parents. The coaches visited the school multiple times before school started and informed the parents about the intervention. The parents who participated in the intervention were given written information about the study related to the intervention. Parents who agreed to participate in this study signed an informed consent.

### School-based intervention

**Fig. 2:**
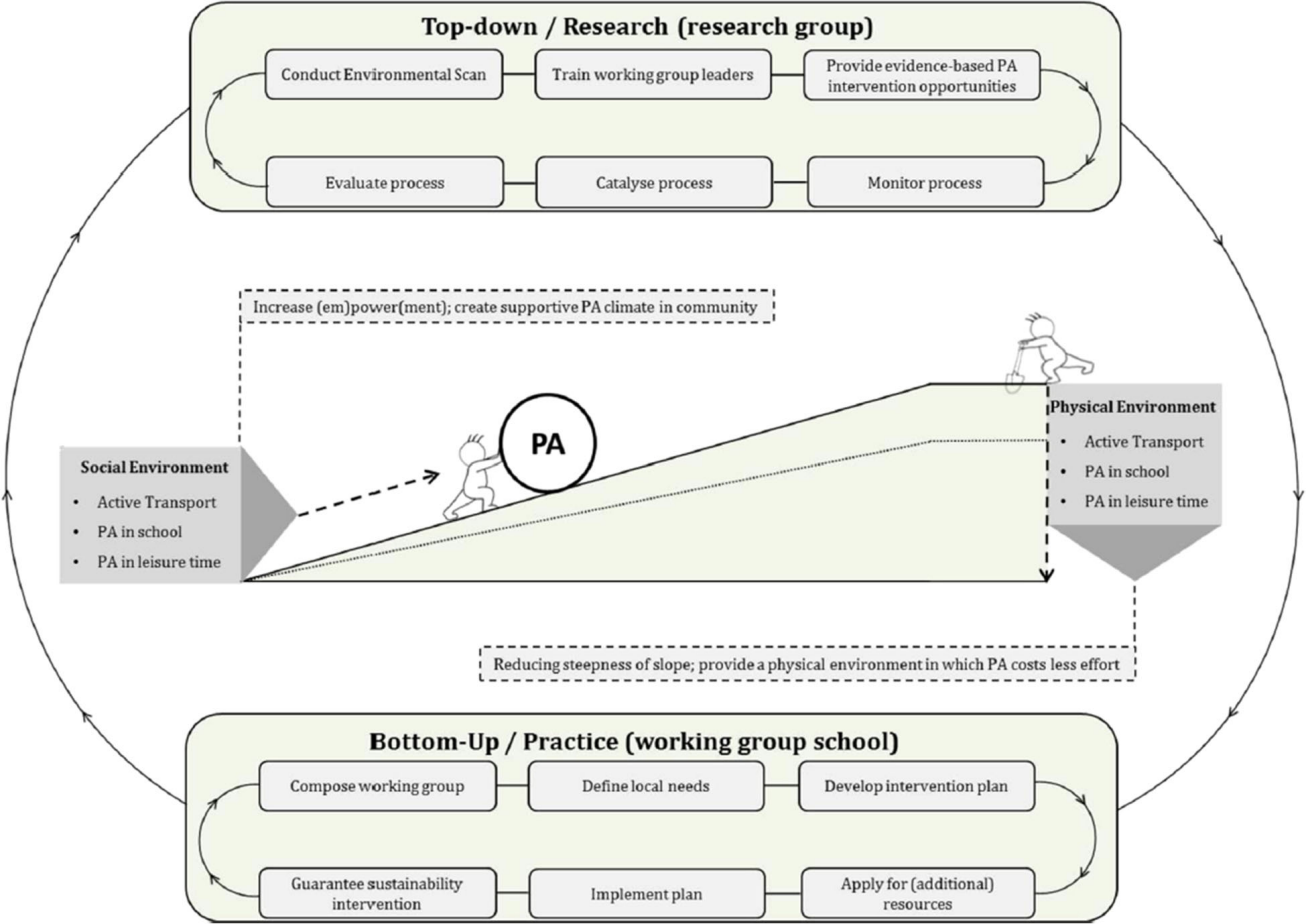
A visual presentation of the mutual adaptation approach [41]

The design of the school intervention follows the combined top-down/bottom-up approach previously successfully applied in the Active Living study (Fig. 2) [39, 41]. In this mutual adaptation approach [26, 42], a steering committee develops the KEIGAAF principles, and KEIGAAF working groups at school level implement these principles in accordance with the needs of the school and its community. These principles are quite generic; the working groups have the autonomy to make almost all decisions regarding the intervention content. An action-oriented approach is applied: the intervention is continuously developed and adapted according to local needs and current systems. Adapting an intervention to local needs enhances intervention effectiveness [43].

**Fig. 3:**
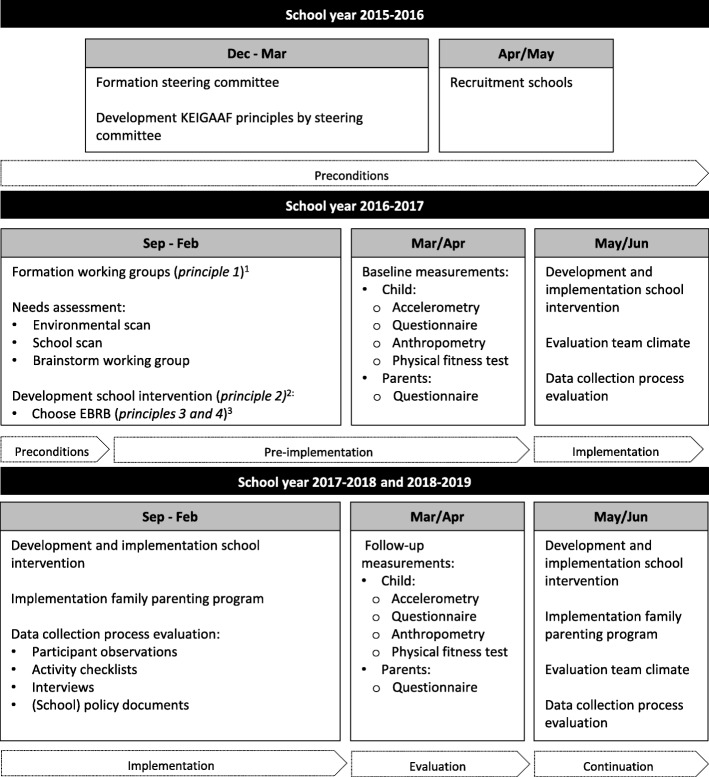
Timeline of KEIGAAF project. ^1^ Principle 1: each school forms a working group, consisting of school staff, local health or sport professionals, a health promotor, and/or the school doctor, and parents. ^2^ Principle 2: The working group members develop and implement the KEIGAAF school intervention according to their pupils’ needs and community possibilities. ^3^ Principle 3 and 4: The intervention’s aims are increasing children’s PA, decreasing sedentariness and improving nutrition behavior. The working groups decide which energy balance-related behaviors they will target primarily

The KEIGAAF principles (see Fig. 3), as developed by the steering committee, are:

1. Each school forms a working group, consisting of school staff, e.g., teachers, the school principal, and/or the PE teacher; local health or sport professionals, such as a social worker; a health promotor; and/or the school doctor; and parents. This “interdisciplinary” team has more knowledge and resources than a working group consisting of school staff only.
2. The working group members collaborate in developing and implementing the KEIGAAF school intervention according to their pupils’ needs and community possibilities. Intervention activities do not necessarily have to be completely new initiatives but can be actions to strengthen and combine existing activities. To develop and implement their own KEIGAAF intervention, the working group members meet regularly. During these meetings, their plans and actions are evaluated and improved.
3. The intervention is aimed at increasing children’s PA, decreasing sedentariness, and improving nutrition behavior. The intervention is primarily designed to affect health behaviors of 7 to 12-year-old children.
4. The working groups decide which EBRB/EBRBs they will target primarily, and the extent and order in which this EBRB is targeted during the project period.

The eight working groups are supported by four trained health promotors from Maastricht University or Fontys University of Applied Sciences. The health promotors advise the working groups on effective interventions and discuss study results with them. They meet regularly with each other and with the steering committee to analyze feedback regarding the intervention implementation and exchange best practices. If appropriate, the best practices are adapted and transferred to other working groups. Besides the personal support, each working group receives a small budget (approximately €2500 for three years) to initiate EBRB-promoting interventions.

### Planning

Schools were recruited in April and May 2016. During the first six months of the next school year (2016–2017), the working groups were formed and local needs defined. To support the working groups in defining their needs, a needs assessment was conducted (see *Data collection*), consisting of: a) a school scan, b) an environmental scan, and c) working group discussions. Based on local needs, iterative KEIGAAF action plans were developed. Implementation of the KEIGAAF interventions started after the baseline measurements were conducted in March and April 2017. Implementation of the KEIGAAF interventions continues until the final measurements at the end of two years (March and April 2019). The aim is to make sure that the KEIGAAF principles become embedded in the school structure and will be sustained after the final measurements.

### Family-based intervention

A family-based parenting program was implemented stepwise from September 2017. The family-based program (Lifestyle Triple P) mainly targets parents whose children are overweight or obese. The aim of the intervention is to improve energy balance-related parenting practices [33, 44]. In eight group sessions and four one-on-one home sessions, Lifestyle Triple P coaches coach parents in positive parenting, nutrition-related parenting practices, and PA-related parenting practices, to support parents in creating a healthy home environment. While the parents attend the Triple P Lifestyle group sessions, their children participate in active fun group play provided by sports students or physical education teachers.

### Data collection

The same data is collected for both the intervention group and the control group. The primary researcher (SV-J) manages the data collection and conducts the measurements in collaboration with trained research assistants. The study is ongoing and data collection has not finished. The data collection is monitored by the Clinical Trial Center Maastricht.

#### Needs assessment

At the start of the project (September to November 2016), an environmental scan and a school scan were conducted. For intervention schools, the outcomes of both scans together with the results of working group discussions on local needs served as a basis for the development of the KEIGAAF school intervention by the working groups. The results of the environmental scan and school scan of the control schools were only used for the process evaluation.

##### Environmental scan

An environmental scan, i.e., the SPACE checklist [45], assessed the PA friendliness of the neighborhoods in which the intervention and control schools were located. The SPACE checklist is a validated and adapted version of the Neighborhood Environment Walkability Scale (NEWS) [46]. The checklist was adapted to the Dutch context [45]. The scan was conducted by a researcher and a research assistant at baseline. The same procedures as described by Van Kann and colleagues [41] were applied. The SPACE checklist will also be completed at the end of the project.

##### School scan

At baseline, the schools’ principal, teachers, and/or the PE teacher were invited to complete a digital school scan. The school scan was based on an evaluation tool developed for primary schools [47]. This tool had already been in use for five years and was refined annually. This scan assessed the school’s activities in the social and physical environment, and school policies, to stimulate PA and healthy nutrition behaviors. Themes are physical education, PA during recess, PA friendliness of schoolyard, PA school activities, active transportation, afterschool PA, PA school policy, teacher and parental involvement in PA promotion at school, nutrition education, healthy nutrition-promoting physical environment, nutrition-promoting activities (including national), nutrition school policy, teacher and parent involvement in healthy nutrition at school. At the beginning of each school year, the school scan is distributed digitally to the schools. The school scan will be completed each year by the same person.

#### Children

Child measurements were collected at baseline (March and April of 2017) and will be collected after one year and two years (March and April 2018 & 2019). Data collection lasts about a week per participating school.

##### Accelerometry

PA and SB are measured via accelerometers (Actigraph GT3X+, Pensacola, FL, USA). The children wear the accelerometers for seven consecutive days. The accelerometers are attached to a belt worn around the child’s hip. The children are instructed to wear the accelerometers all day during waking hours but to remove them before performing water-based activities, such as swimming and showering.

##### Questionnaire

Children complete a questionnaire individually in the classroom. The questionnaire was pretested among children of the same age attending different schools to ensure that the questions were clear and understandable. The questionnaire assesses demographics, PA at school, sports participation, NB, PA enjoyment, and food, drink, and PA preferences and is for the most part based on validated questionnaires (Table 1).

**Table 1:** Concepts, number of items, and example question of the child questionnaire

Concept	N items	Items
Demographics	12	Name of school, grade (4th, 5th, 6th), birth month, birth year, gender, home address, country of birth, country of birth parents, number of siblings
PA on a school day	6	Television viewing, transportation to school and from school (walking, cycling, by step/waveboard/skateboard/skates, driven by scooter, driven by car, driven by bus, other), longest performed activity during recess (running, jumping, playing, walking, sitting and chatting, standing and chatting, playing inside, other), activity after school time yesterday (playing outside, sports, active play inside, low intensity play inside, swimming, music or drama lesson, PC/tablet/smartphone use, watching television)
Sports participation	1	Member of sports clubs (no, soccer, tennis, dancing, gymnastics/ballet, swimming, horse riding, martial art, basketball, volleyball, handball, hockey, badminton, athletics, cycling, table tennis, korfball, scouts, afterschool PA, other)
Nutrition intake on a school day [77]	12	Breakfast intake, fruit intake and vegetable intake at school (yes/no), amount of fruit intake (0.5, 1, 2, 3 or more pieces), water consumption at school yesterday (yes/no), sugar-sweetened beverages (yes/no), milk drinks (yes/no), energy drinks (yes/no), and sports drinks consumption at school (yes/no), candy (yes/no), cookie (yes/no), and snack consumption (yes/no)
PA enjoyment [78]	16	Example: When I am physically active… I enjoy it. (5-point Likert scale: 1 = totally disagree, 4 = totally agree, 5 = don’t know)
Food and drink preferences [79]	16	Examples: Which of the following foods do you prefer more? (fruit or savory snacks) / Which of the following drinks do you prefer more? (soft drink or fruit juice)
PA preferences [79]	28	Example: Which of the following physical activities do you prefer more? (cycling or watching television)

##### Anthropometry

Anthropometric measurements are taken during a physical education (PE) lesson. Standing height, weight, and waist circumference are measured by researchers or research assistants according to a standardized protocol. Shoes have to be taken off. The children are weighed with a digital weighing scale to the nearest 0.1 kg (Seca weighing scale 803). Height is measured with a stadiometer to the nearest 0.1 cm (Seca 213). Weight and height are used to calculate the Body Mass Index (BMI), which is adjusted for age and gender (BMI z-scores) using the values of a Dutch reference population [48]. Waist circumference is measured with a measuring tape to an accuracy of 0.1 cm (Seca 201). The measuring tape is placed around the umbilicus [49].

##### Physical fitness test

The 15 m endurance shuttle run test, known as the Progressive Aerobic Cardiovascular Endurance Run (PACER), is used to assess the children’s aerobic fitness [50]. The PACER is an item of the EUROFIT test battery [51]. The test is administered during the same PE lesson in which the children’s anthropometric measurements are taken. The assessment of the test lasts between 10 and 15 min. The physical fitness test is not conducted during the time that the children wear the accelerometer.

#### Parents

Parents are requested to fill in a questionnaire during the one-week measurement period at baseline and at follow-up (March and April 2017, 2018 & 2019).

##### Questionnaire

The parent questionnaires are handed to the children, and the parents complete them at home. The questionnaires assess demographics, parent’s PA pattern routines, PA parenting practices, family health climate, child’s NB, anthropometry, and parental school involvement. At follow-up, parents of the intervention schools also receive evaluation questions concerning the KEIGAAF intervention (Table 2).

**Table 2:** Concepts, number of items, and example question of the school-based parent questionnaire

Concept	N items	Items
Demographics	22	Name of school child, child’s grade (4th, 5th, 6th), gender of child, birth date of child, birth date of parent, birth date of partner, postal code, relation to child (mother, stepmother, father, stepfather, guardian, other), country of birth, highest education level (no education, primary school, pre-vocational school, secondary education, lower vocational education, higher vocational education, university), hours per week of paid employment (no paid job, 16 or less, 17–24, 25–32, more than 32 h), member of sports club (yes/no), family situation (living together with a partner, single, other) / *Partner*: relation to child, country of birth, highest education level, hours per week of paid employment, member of sports club / number of children (1, 2, 3, 4 or more), number of cars (1, 2, 3 or more).
Parents’ PA transportation routines (PATRns) [80]	4	Example: If I have to go somewhere nearby, I am always inclined to take the bike or to go on foot. (5-point Likert scale: 1 = completely disagree, 5 = completely agree)
PA parenting practices [81]	6	Parental logistic support and restrictions on access to sedentary activities. Example: I enroll my child in sports teams and clubs such as soccer, basketball, and dance. (4-point Likert scale: 1 = completely disagree, 4 = completely agree)
Family health climate [82]	31	Family climate regarding PA and nutrition. Example: In our family… a healthy diet plays an important role in our lives. (4-point Likert scale: 1 = completely disagree, 4 = completely agree)
Child’s nutrition behavior [77]	25	Breakfast consumption, fruit intake, vegetable intake, candy intake, cookie intake, snack intake (0 to 7 days; amount of portions per day), sugar-sweetened beverages consumption, light soda consumption, fruit juice consumption, sweet milk drinks consumption, milk consumption, water and tea without sugar consumption (0 to 7 days; amount of glasses per day)
Anthropometry	3	Weight and height of child, weight and height of parent, weight and height of partner.
Parental school involvement	5	Contact with teacher (5-point Likert scale: 1 = never; 5 = every week), assisting school with school activities (5-point Likert scale: 1 = never; 5 = always), attending parental meeting (5-point Likert scale: 1 = never; 5 = always), feeling welcome at school (5-point Likert scale: 1 = not welcome; 5 = welcome), reading school information (5-point Likert scale: 1 = never; 5 = always).
KEIGAAF evaluation (added at follow-up) *only for intervention group*	6	Aware of KEIGAAF (not aware, heard of it, fully aware), general impression of KEIGAAF (5-point Likert scale: 1 = very bad; 5 = very good), involvement in KEIGAAF (5-point Likert scale: 1 = very little; 5 = very much), experiencing changes at school as a result of KEIGAAF (5-point Likert scale: 1 = very little; 5 = a lot), experiencing changes at home as a result of KEIGAAF (5-point Likert scale: 1 = very little; 5 = a lot), importance of KEIGAAF (5-point Likert scale: 1 = very unimportant; 5 = very important)

##### Questionnaire on family-based intervention

The questionnaire related to the family-based intervention is filled in at the start and at the end of the intervention (after three months) by participants of the Lifestyle Triple P intervention. The questionnaire assesses general parenting practices [52], nutrition parenting practices [53, 54], and PA parenting practices [13, 55, 56]. Questions to evaluate and improve the program are included in the follow-up questionnaire.

### Process evaluation

An elaborate process evaluation will be conducted in which the processes involved in the implementation of KEIGAAF and contextual factors influencing these processes are illuminated. For this, a mixed-methods approach will be used: 1) an activity checklist is filled in throughout the implementation period to gain insight into what is implemented by the different KEIGAAF working groups; 2) a quantitative team climate checklist is filled in at baseline, after one and after two years. This checklist provides information about the climate for innovativeness within a working group [57, 58]; 3) school policy documents are collected, providing information about policies influencing intervention implementation; 4) participant observations of the working groups and semi-structured interviews with working group members are conducted to gain additional knowledge about factors inhibiting or facilitating implementation; and finally, 5) the data of the environmental scan and the school scan provide insight into neighborhood and school factors impeding or facilitating intervention success. Data is collected throughout the implementation period of the combined school- and family-based interventions. The timeline of the KEIGAAF project is shown in Fig. 3.

### Data analysis

Descriptive statistics will be used for baseline data. Accelerometer data will be analyzed using Actilife (Version 6.13.3). To correct for nesting of the data, multilevel regression analyses will be used to analyze it. When studying intervention effects, baseline data will be taken into account as possible confounders. Using forward selection, the random and fixed effects part will be further refined by likelihood ratio tests. Intervention effectiveness will be tested at T1 (after one year) and at T2 (after two years). A two-sided test with a type I error rate of 5% will be used. Statistical analyses will be conducted using SPSS 24.0 (IBM Corp, USA).

Quantitative process data will be analyzed with SPSS. Inductive content analysis will be performed to analyze the qualitative process data. Interviews will be transcribed verbatim, and themes and concepts arising from the data will be coded. Outcomes of the process evaluation will be linked to the outcomes of the effectiveness study to clarify the effects on the primary and secondary outcome measures.

### Power calculation

Children’s health outcome, i.e., BMI z-score, is the primary outcome measure in this study, while interventions are targeted on more distal health behaviors (EBRBs). EBRBs are used as additional outcome measures and considered as predictors of BMI z-scores. Therefore, baseline measurements of the participants’ BMI z-score were used to calculate the power of the study. At baseline, 419 intervention children and 104 control children were included in the study. Children were in grade 4, 5, or 6. In two-level, school-based, cluster randomized trials with BMI z-score as the outcome, the intra-class correlation (ICC) was mostly 0.03 or smaller (e.g., [59–61]). Taking a three-level design into consideration, it is assumed that the ICC ranges from 0.005 for the school level and 0.025 for the class level to an ICC of 0.025 for the school level and an ICC of 0.005 for the class level. For the given sample size, the smallest detectable difference in BMI z-score after two years of intervention ranges between 0.38 and 0.44 when the power is 80%, and between 0.44 and 0.51 when the power is 90%. Using Lipsey’s [62] cut-off scores, this indicates a moderate effect. Empirical results show that differences of this size are attainable for combined school- and family-based interventions [63, 64]. Although the effects are moderate, they may lead to clinical significant effects at the population level if they are sustained for several years [65].

## Discussion

In this paper, we present the design of a quasi-experimental study in which we conduct an effect and process evaluation of a context-based, school- and family-based PA and nutrition intervention on children’s BMI z-score, their EBRBs, and physical fitness after one and after two years. The focus on two important environmental settings within the child’s environment, i.e., school and family, is considered a strength of this intervention. Ecological models, such as the socio-ecological model of Bronfenbrenner, theorize the importance of targeting multiple systems because of the interdependence of these systems [66]. Furthermore, systematic reviews on school-based interventions with parental involvement have shown the importance of targeting both settings [67, 68]; school-based interventions targeting more home-related factors seem more likely to be effective [68].

The intervention is developed by the intervention schools in collaboration with local professionals and parents, which is in line with the Health Promoting Schools (HPS) framework (also known as Coordinated School Health (CSH) [29, 69]) [70]. The HPS approach has been proven to be effective in improving children’s EBRBs [25, 71]. Because of the combined bottom-up and top-down development of the KEIGAAF intervention, it is expected to be an effective and sustainable version of the HPS: the bottom-up development ensures that the intervention fits the local situation, while top-down decisions (for example, policy decisions taken by the school board) actively stimulate schools to take action, and thereby accelerate the process of actions being taken [72].

In addition to an effect evaluation, an extensive process evaluation will be conducted to provide insights into the processes of designing and implementing the interventions and the role of the context. Few school-based intervention studies have looked at intervention effectiveness and its implementation [73], although this latter information and the link between effectiveness and implementation are important for scaling an intervention and developing future health promotion interventions.

To measure intervention effectiveness, the study has a long duration, and multiple data collection tools are used to measure the different outcomes. The use of accelerometers to measure PA and sedentary behavior is advocated [74] because physical activity questionnaires are subject to under- or over-reporting due to recall bias or social desirability [75]. The questionnaires to measure the other outcome measures are based on validated questionnaires.

This study also has some limitations, e.g., only three schools were recruited as control schools. In general, schools are hesitant to participate in additional projects because of the time pressure experienced. Additionally, recruiting an appropriate control group was difficult: there are numerous national and local efforts to prevent overweight and obesity among children in the Netherlands (e.g., EPODE, healthy schools). It should be noted that the schools located in Maastricht are subject to local health-promoting initiatives. To be able to define the potential effectiveness of the KEIGAAF approach, PA and nutrition-promoting activities implemented in the control schools will also be monitored.

A final limitation is possibly the use of a quasi-experimental study design. A randomized controlled trial is considered the gold standard for studying intervention effectiveness. However, in a project in which there is no strict control over what is implemented and how the system influences implementation, an RCT design is considered inappropriate [76]. Therefore, we consider a quasi-experimental study a good option to study intervention effectiveness.

## Additional file

Additional file 1: SPIRIT Checklist (DOC 120 kb)

## Abbreviations

BMI: Body mass index
CHS: Comprehensive school health
EBRB: Energy balance-related behaviors
EPODE: Ensemble Prévenons l’Obésité Des Enfants
HPS: Health promoting schools
KEIGAAF: Kansen in EIndhoven voor GezinsAAnpak met Fontys
NB: Nutrition behavior
PA: Physical activity
PE: Physical education
SB: Sedentary behavior
SES: Socio-economic status
